# Subjective socioeconomic status is associated with risk aversion in a community-based cohort of older adults without dementia

**DOI:** 10.3389/fpsyg.2022.963418

**Published:** 2022-11-07

**Authors:** Gali H. Weissberger, S. Duke Han, Lei Yu, Lisa L. Barnes, Melissa Lamar, David A. Bennett, Patricia A. Boyle

**Affiliations:** ^1^Interdisciplinary Department of Social Sciences, Bar-Ilan University, Ramat Gan, Israel; ^2^Department of Family Medicine, USC Keck School of Medicine, Alhambra, CA, United States; ^3^Department of Psychology, USC Dornsife College of Letters, Arts, and Sciences, Los Angeles, CA, United States; ^4^USC School of Gerontology, Los Angeles, CA, United States; ^5^Department of Neurology, USC Keck School of Medicine, Los Angeles, CA, United States; ^6^Rush Alzheimer’s Disease Center, Rush University Medical Center, Chicago, IL, United States; ^7^Department of Neurological Sciences, Rush University Medical Center, Chicago, IL, United States; ^8^Department of Psychiatry and Behavioral Sciences, Rush University Medical Center, Chicago, IL, United States

**Keywords:** risk aversion, older adults, subjective socioeconomic status, education, income

## Abstract

Attitudes towards risk impact financial decisions that are critical in older adulthood. Socioeconomic status (SES) influences an individual’s level of risk aversion; however, the association of subjective SES (i.e., social standing relative to others) with risk aversion has not been explored. We examined whether subjective SES is associated with risk aversion independent of objective SES (i.e., income, education). Participants were 933 older adults without dementia from the Rush Memory and Aging Project (MAP) or Minority Aging Research Study (MARS), two longitudinal epidemiologic studies of aging. Participants completed assessments of risk aversion, subjective SES, and cognition. We examined associations of subjective SES with risk aversion using mixed models adjusting for participant characteristics, objective markers of SES and global cognition. In bivariate analyses, lower global cognitive functioning, lower income, female sex, Black race, and lower subjective SES were associated with greater risk aversion. Results of the nonlinear mixed effects model revealed that higher subjective SES was associated with less risk aversion (Estimate = −0.238, SE = 0.083, *p* = 0.004), after controlling for covariates. Age, sex, race, and global cognition were also associated with risk aversion in the mixed effects model (*p*s ≤ 0.03), although income and education were not (*p*s ≥ 0.27) The relationship between subjective SES and risk aversion did not differ by sex or race (*p*s ≥ 0.31). Findings suggest that subjective SES contributes to risk aversion regardless of sex or race. Findings support the importance of considering subjective indicators of SES as they may impact an older adult’s economic preferences.

## Introduction

Attitudes towards risk influence important financial decisions that are important throughout the lifespan and critical in older adulthood, such as how to invest savings, distribute and transfer wealth, and allocate assets, thereby directly impacting financial wellbeing. Risk aversion, or preference for low-yield but more certain financial options over high-yield but less certain financial options, has been associated with negative financial outcomes (Finke and Huston, 2003) such as lower net worth and less retirement savings (Bernheim et al., 2001; Finke and Huston, 2003; Watson and McNaughton, 2007), as well as poor financial and healthcare decision making among older adults (Boyle et al., 2012). Thus, understanding the determinants of risk aversion in old age is imperative for maximizing the financial wellbeing of this age group.

It is well-documented that objectively measured socioeconomic status (SES) influences an individual’s level of risk aversion (Bajtelsmit and Bernasek, 2001; Kish-Gephart, 2017; Amir et al., 2018). Numerous studies have found that individuals with less income are more risk averse than individuals with more income (Riley and Chow, 1992; Grable, 2000; Haushofer and Fehr, 2014). Other aspects of SES, such as education and occupation level, have also been associated with risk aversion, with studies finding that increased educational attainment and higher occupational status are related to less risk aversion (Grable, 2000; Outreville, 2015). Although few studies have investigated these associations specifically in older adults, likely due to the challenges of recruiting older adults participants (Mody et al., 2008), some evidence indicates that objective indicators of SES may also be related to risk aversion among older adults (Barsky et al., 1997, Schurer, 2015; but see our previous work, Boyle et al., 2011).

In addition to objectively measured SES, recent studies suggest that an individual’s subjective impression of his or her social standing may provide valuable information above and beyond objective measures of SES. For example, higher subjective SES has been shown to be associated with better health outcomes, including physical health and psychological wellbeing, even after adjusting for objective indicators of SES (Adler et al., 2000; Singh-Manoux et al., 2005; Demakakos et al., 2008; Cundiff and Matthews, 2017). Thus, subjective SES seems to offer utility in understanding variability in physical health across individuals. It stands to reason that subjective SES may also provide additional utility in understanding variability in risk aversion in old age. In support of this, Sheehy-Skeffington (2020) discuss in a review that individuals must navigate their environment in the presence of others with whom they cooperate with or compete against; thus, it is likely that decision making is shaped not only by one’s absolute resources, but also by one’s relative resources in comparison to others. With regard to risk aversion, it has been shown that having a higher sense of power (i.e., the capacity to influence others), associated with high social status, leads to greater risk taking behaviors possibly because it increases confidence that the future will manifest as planned (Anderson and Galinsky, 2006). Thus, lower perceived social status may lead to increased risk aversion *via* a lower sense of power and control over future events. However, to our knowledge, the association of subjective SES with economic preferences such as risk aversion has yet to be explored.

In this study, we examined whether subjective SES is associated with risk aversion independent of objective SES among non-demented participants from two community-based cohort studies of aging. Subjective SES was measured *via* a questionnaire that asked participants to indicate where they believe they stand on a 10-rung ladder of social standing relative to others in their community and separately relative to the United States population more generally. Risk aversion was assessed utilizing standard behavioral economics questions in which participants were asked to choose between a guaranteed payout or a gamble that would increase the payout or result in no payout. Given some research suggesting an association between lower objective SES and more risk aversion (e.g., Schurer, 2015), we hypothesized that lower subjective rankings of social standing would be associated with greater risk aversion, after controlling for demographic covariates and objective markers of SES including education and income. We also investigated potential moderating effects of sex and race, given evidence that these factors moderate the relationship between subjective SES and physical health (Demakakos et al., 2008; Cundiff and Matthews, 2017).

## Materials and methods

### Participants

Participants were 933 older adults without dementia from the Rush Memory and Aging Project (MAP) or Minority Aging Research Study (MARS) recruited from community organizations, subsidized housing, and local residential facilities (e.g., retirement homes, senior housing facilities) in the Chicago metropolitan area. The MAP study is a cohort study of aging and dementia that began in 1997 (Bennett et al., 2018). MARS is a cohort study of decline in cognitive function and risk of Alzheimer’s disease in older African Americans that began in 2004 (Barnes et al., 2012). All MAP and MARS procedures (harmonized for combined investigations like this one) have been approved by the Rush University Medical Center institutional review board, and procedures were conducted in accordance with the Declaration of Helsinki. All participants provided written informed consent prior to study participation, and signed a repository consent allowing their data to be repurposed. More information on access to the data can be found on the Rush Alzheimer’s Disease Center website.^1^

A sub-study of decision making was introduced into MAP in 2010, and into MARS in 2018. When the present analyses were conducted, 1,012 participants completed the decision making sub-study and had data available for analyses. Sixty-five individuals with dementia were excluded from analyses. Fourteen additional participants indicated a race/ethnicity other than White, non-Hispanic or Black and were thus excluded from analyses. This left a total sample of 933 participants who had complete data for these analyses.

### Assessment of risk aversion

Risk aversion was assessed using a standard behavioral economics approach consistent with previous work by our group (Boyle et al., 2011, 2012; Han et al., 2012). Briefly, participants were asked if they would prefer a certain $15 payout (safe payment), or a coin toss (gamble) in which they could potentially get an amount greater than $15 if heads is flipped, and nothing if tails is flipped. There were 10 items, each posing gain amounts that randomly varied between $20 to $300. Questions were designed in such a way that any gamble that offers a potential gain of $30 results in the same long run average or expected utility as the safe payment, but any potential gamble gain over $30 exceeds the expected value of the safe payment.

### Composite score of subjective socioeconomic status (SES)

**Figure 1:**
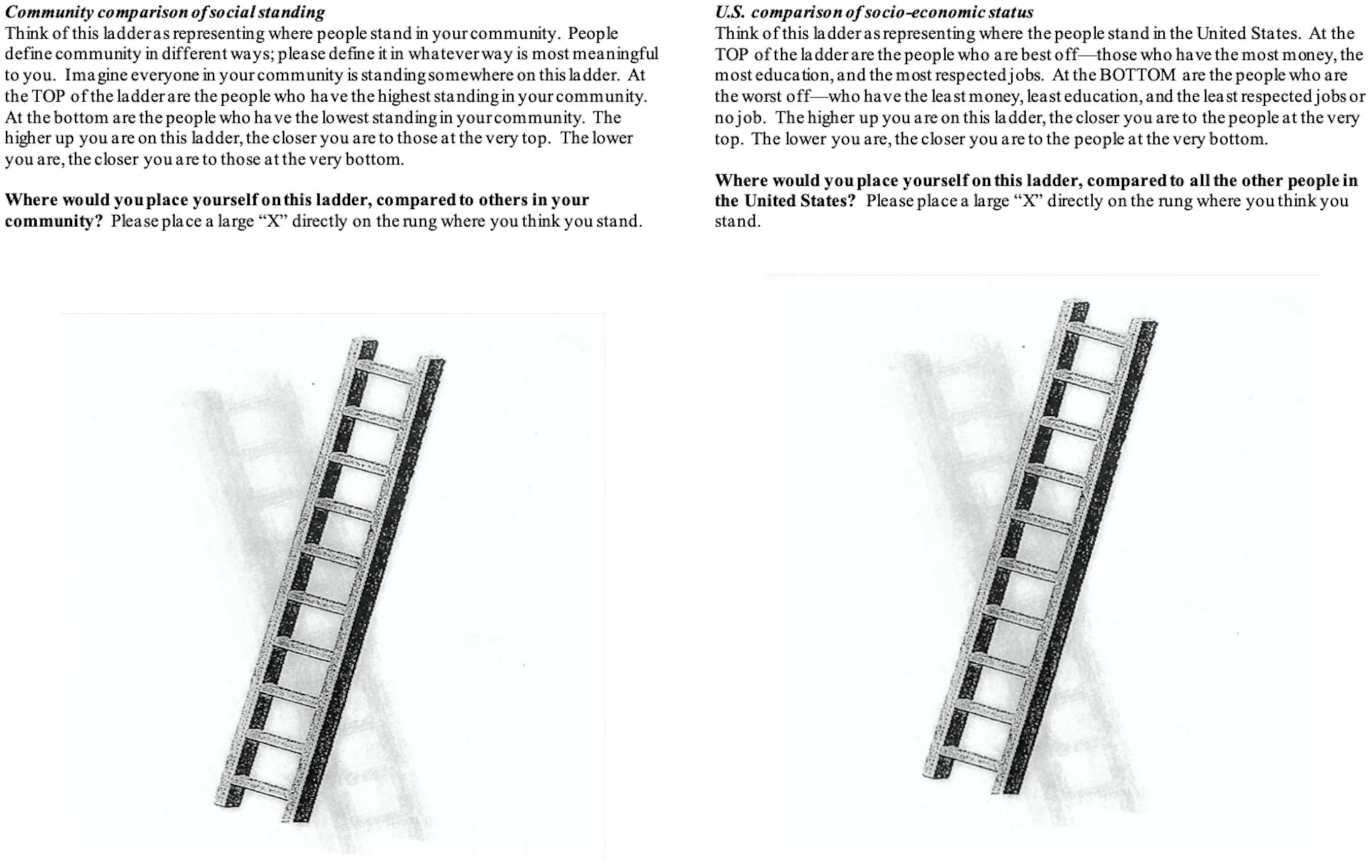
The two items of self-perceived SES used in this study. Items were adapted from the MacArthur scale of subjective social status.

Subjective SES is a composite measure of two items of self-perceived SES (Figure 1) adapted from the MacArthur Scale of Subjective Social Status (Adler and Stewart, 2007). The scale assesses a summative impression of social status indicators using two pictorial images of a ladder (Adler and Stewart, 2007). The first ladder item presents an image of a ten-rung ladder and asks participants where they would place themselves on the ladder relative to others in their community; the first ladder item specifies that the top of the ladder are those who have the highest standing in the community, and the bottom of the ladder are those who have the lowest standing. The second ladder item asks participants where they believe they stand compared to all other people in the United States. The item specifies that the top of the ladder are those who have the most money, the most education, and the most respected jobs. Those at the bottom of the ladder have the least money, least education, and least respected jobs or no job. For each ladder item, participants are asked to place an “X” directly on the rung where they think they stand relative to others. Each rung is assigned a value of 1 through 10, with the topmost rung given a score of 10 and representing the highest social standing. The mean of the two ladders scores is calculated to derive a composite score of subjective SES. Higher mean values indicate self-perceived standings closer to people at the very top in terms of money, education, and occupation. Subjective SES has been related to employment grade, education, household and personal income, household wealth, and other objective indicators of SES (Singh-Manoux et al., 2005; Adler et al., 2008) and a range of health outcomes (see Adler and Stewart, 2007 for summary).^2^ The MacArthur scale of subjective social status has been shown to have moderate to good concurrent validity and deemed to have face validity (Ferreira et al., 2018), as well as strong construct validity (Cundiff et al., 2013).

### Objective indicators of SES

Objective SES was measured using education and income, two commonly utilized metrics of objective SES (Cohen et al., 2008; Demakakos et al., 2008). Participants self-reported years of education at their baseline cognitive assessment based on the number of years of regular school completed. Income was acquired during each participant’s baseline assessment using a single question asking participants to select one of 10 levels of total family income ($0–$4,999; $5,000–$9,999; $10,000–$14,999; $15,000–$19,999; $20,000–$24,999; $25,000–$29,999; $30,000–$34,999; $35,000–$49,999; $50,000–$74,999; $75,000 and over). Scores range from 1 to 10, with 10 indicating the highest income bracket.

### Assessment of cognition

A comprehensive neuropsychological assessment of 21 measures was administered to all participants. Details of the measures can be reviewed in Table 1. Nineteen of the 21 measures are used to derive a cognitive composite score, with two measures, The Complex Ideational Material test and the Mini Mental Status Examination (MMSE) used for descriptive and clinical purposes only. The measures included in the composite score were word list memory (total number of words recalled immediately after the three learning trials), word list recall (total words recalled after a delay), and word list recognition from the Consortium to Establish a Registry for Alzheimer’s Disease (CERAD) battery, immediate and delayed recall conditions of Logical Memory Story A and the East Boston Story, verbal fluency (animals, fruits/vegetables), the Boston Naming Test, the National Adult Reading Test, forward and backward conditions of the Wechsler Memory Scale-revised Digit Span subtest, Digit Ordering, the Symbol Digit Modalities test, Number Comparison, the Judgment of Line Orientation test, Standard Progressive Matrices, and Stroop color naming and word reading conditions. The raw scores from each of the 19 measures used to derive a cognitive composite score (Wilson et al., 2002, 2003) were converted to z-scores using baseline mean and standard deviation of the entire MAP and MARS cohorts. Each participant’s 19 z-scores were then averaged to derive a global cognition composite score, as has been published previously (e.g., Wilson et al., 2002, 2003; Bennett et al., 2005; Fleischman et al., 2005; Schneider et al., 2012; Bennett et al., 2012a,b; Boyle et al., 2013; Han et al., 2016).

**Table 1:** Description of neuropsychological tests included within the cognitive composite score.

Test	Description	Scoring Details	Use
The Complex Ideational Material Test	Receptive language	Number of items correctly answered	Descriptive/clinical purposes
Mini Mental Status Examination	Cognitive screening measure	Number of items correctly answered	Descriptive/clinical purposes
CERAD Word List Memory immediate recall	Immediate recall	12-item list of words; total items recalled after three learning trials	Cognitive composite score
CERAD Word List delayed recall	Delayed recall	Total words from the 12-item list recalled after 30-min delay	Cognitive composite score
CERAD Word List Recognition	Delayed recognition memory	Total words identified as belonging to the 12-item word list	Cognitive composite score
Logical Memory Story A immediate recall	Immediate recall	Total details recalled immediately after hearing story	Cognitive composite score
Logical Memory Story A delayed recall	Delayed recall	Total details recalled after a 30-min delay	Cognitive composite score
East Boston Story immediate recall	Immediate recall	Total details recalled immediately after hearing story	Cognitive composite score
East Boston Story delayed recall	Delayed recall	Total details recalled after a 15-min delay	Cognitive composite score
Verbal Fluency (animals, fruits/vegetables)	Semantic knowledge; rapid word generation	Total words generated within 60-s	Cognitive composite score
Boston Naming Test	Confrontation naming and lexical retrieval abilities	Total items correctly identified	Cognitive composite score
The National Adult Reading Test	Reading ability	Total words correctly read and pronounced	Cognitive composite score
WMS-R Digit Span subtest, forward condition	Auditory attention and working memory	Longest string of numbers recalled in forwards order	Cognitive composite score
WMS-R Digit Span subtest, backward condition	Auditory attention and working memory	Longest string of numbers recalled in backwards order	Cognitive composite score
WMS-R Digit Ordering subtest	Auditory attention	Number of digits correctly recalled in ascending order	Cognitive composite score
WMS-R Symbol Digit Modalities test	Complex scanning, visual tracking, and perceptual speed	Number of numbers correctly written to match specific symbols	Cognitive composite score
WMS-R Number Comparison	Complex scanning, visual tracking, and perceptual speed	Test of complex scanning and visual tracking	Cognitive composite score
The Judgment of Line Orientation Test	Ability to estimate spatial relationships	Correct number of spatial matches across 30 items	Cognitive composite score
Standard Progressive Matrics	Non-verbal abstract reasoning	Number of correctly completed patterns	Cognitive composite score
Stroop Word Reading	Processing speed	Number of words correctly read	Cognitive composite score
Stroop Color Naming	Cognitive inhibition	Number of colors correctly named	Cognitive composite score

CERAD = Consortium to Establish a Registry for Alzheimer’s Disease; WMS-R = Wechsler Memory Scale Revised.

### Clinical diagnoses

A clinician with expertise in aging and dementia diagnoses diagnosed participants with dementia in accordance with NINCDS/ADRDA criteria (McKhann et al., 1984), as previously described (Bennett et al., 2006). Briefly, diagnostic classification occurred in a three-step process. First, an education-adjusted rating of impairment was applied to 11 neuropsychological tests commonly used for clinical classification of Alzheimer’s disease. Second, to determine presence of cognitive impairment, a neuropsychologist reviewed results from the battery of cognitive tests along with participant background information including education, occupation, sensory and motor deficits, and effort. The neuropsychologist rendered a clinical judgment regarding the presence of cognitive impairment or dementia. Third, a physician with experience in evaluating older persons with and without dementia reviewed all available data, including the neuropsychologist’s impressions and the raw neuropsychological test data, interviewed and examined the participant, and also rendered a clinical judgment regarding whether the participant has experienced cognitive decline, and whether there is evidence of stroke, depression, or other common conditions that may contribute to dementia. These clinical judgments were entered into a laptop computer and an actuarial decision tree generated seven clinical diagnoses. To receive a diagnosis of dementia, meaningful decline in cognitive function and impairment in multiple areas of cognition must be observed, in accordance with NINCDS/ADRDA criteria. Mild Cognitive Impairment refers to individuals who are rated as impaired on cognitive testing by the neuropsychologist but not demented by the examining clinician. Further details regarding clinical diagnoses can be reviewed in Bennett et al. (2006).

### Demographic covariates

Age is calculated based on birthdate. Race (White or Black) and sex were self-reported.

### Statistical analyses

Based on a commonly used approach in behavioral economics studies (Barsky et al., 1997; Harrison et al., 2007; Harrison and Rutström, 2008; Boyle et al., 2011, 2012), the risk aversion index γ (gamma) was estimated from participant responses to all 10 risk aversion items. Items consisted of safe payoff or gamble payoff options. The gamble payoff for *i*th participant at *j*th question, GP*_ij_*, was defined as,


$$GP_{ij}=\frac{0.5\;\times \;{\mathrm{Gain}}_{j}^{1-\gamma_{i}}}{1-\gamma_{i}}$$

where Gain*_j_* was the gamble gain for the *j*th question, and γ*_i_* was the risk aversion parameter for the *i*th participant. The safe payoff option for the *i*th participant at *j*th question, *SP*_ij,_ was defined as,


$$SP_{ij}=\frac{\;{\mathrm{Safe}}^{1-\gamma_{i}}}{1-\gamma_{i.}}$$

where ‘Safe’ was the safe gain ($15), consistent across all questions.

The probability of subject *i* choosing the gamble option at question *j*, or $P\left(Y_{ij}=1\right)$ , assumes to be dependent on the difference between *GP_ij_* and *SP_ij,_* which is linked through a logistic function,


$$\mathrm{logit}\left(P\left(Y_{ij}=1\right)\right)=GP_{ij}-SP_{ij}$$

Substituting *GP_ij_* and *SP_ij_,* we have,


$$\mathrm{logit}\left(P\left(Y_{ij}=1\right)\right)=\frac{0.5\;\times \;{\mathrm{Gain}}_{j}^{1-\gamma_{i}}-{\mathrm{Safe}}^{1-\gamma_{i}}}{1-\gamma_{i}}$$

where, *Y_ij_* is the item response from the *i*th participant to the *j*th question. To obtain the risk aversion estimate for each individual participant, we maximized the likelihood function constructed based upon a nonlinear mixed effect model. Participants were included as a random effect which was assumed to have a normal distribution.

All continuous variables were assessed for normality and outliers. Given the distribution of gamma, we chose to utilize the summary measure of gamma for bivariate analyses and item level responses for the multivariable analyses. We first conducted bivariate associations (Spearman’s rank order correlations; Wilcoxon two-sample tests) of subjective SES, demographic variables, and global cognition with risk aversion. To investigate the relationship between subjective SES and risk aversion, we expanded the mixed effect model by further modeling γ*_i_* as a function of subjective SES. Age, sex, race, education, income, and cognition were also included as covariates.

## Results

Participant characteristics are presented in Table 2. The sample was mostly female, mostly of White race, and well-educated.

**Table 2:** Distribution of participant characteristics and subjective SES composite score (*N* = 933).

	*Mean*	*SD*
Age	80.84	7.37
Education (years)	15.51	3.02
Sex (% female)	76.63%	–
Race (% Black)	23.37%	–
Global cognition composite	0.28	0.52
Income	7.41	2.37
Subjective SES composite	6.62	1.35
Risk Aversion Coefficient	0.31	0.30

SD = standard deviation; SES = subjective socioeconomic status.

Table 3 displays the distribution of participants who took the gamble for each of the 10 risk aversion items. The percentage of participants in the sample who took the gamble steadily increased as the payouts increased. In brief, approximately 40% of participants took the gamble when the potential payout gain was double ($30) the amount of the safe payout. About 75% of the sample took the gamble when the potential payout gain was six times ($90) the amount of the safe payout; this increased to 85% of the sample when the potential payout gain was 20 times ($300) the safe payout.

**Table 3:** Distribution of participants in the sample (*N* = 933) who decided to take the gamble on the risk aversion task separately for each of the 10 items/gain amounts.

Gain amount	Took gamble	
	*n*	%
20	161	17.26
30	364	39.01
35	452	48.45
45	531	56.91
65	542	58.09
90	699	74.92
110	760	81.46
150	781	83.71
230	801	85.85
300	802	85.96

### Bivariate associations of risk aversion with subjective SES, demographic variables, and cognition

Age was positively correlated with risk aversion (Spearman’s *rho* = 0.06, *p* = 0.050). Education (in years; Spearman’s *rho* = −0.11, *p* < 0.001), income (Spearman’s *rho* = −0.10, *p* = 0.002), global cognitive composite score (Spearman’s *rho* = −0.10, *p* = 0.002), and subjective SES (Spearman’s *rho* = −0.13, *p* < 0.001) were inversely correlated with risk aversion. Males were significantly less risk averse than females (Males: *M* = 0.23, *SD* = 0.26; Females: *M* = 0.34, *SD* = 0.30; Wilcoxon *Z* = −4.20, *p* < 0.0001), and White participants were significantly less risk averse than Black participants (White: *M* = 0.30, *SD* = 0.29; Black: *M* = 0.39, *SD* = 0.31; Wilcoxon *Z* = 3.26, *p* < 0.001).

### Association of subjective SES and risk aversion

Results of the nonlinear mixed effects model revealed that higher subjective SES was associated with less risk aversion, after controlling for age, sex, race, objective SES (i.e., education and income), and global cognitive scores. Age, sex, race, and global cognition were also significantly associated with risk aversion in the mixed effects model, although income and education were not. Results can be viewed in Table 4. Although sex and race were both independent predictors of risk aversion, the interactions between sex and race and subjective SES were not significant, indicating that they did not moderate the relationship between subjective SES and risk aversion (both *p*s ≥ 0.31).

**Table 4:** Results of the nonlinear mixed effects model examining the association of subjective socioeconomic status (SES; bolded in table) with risk aversion (Model 1). Additional models examined whether sex (Model 2) or race (Model 3) modified the relationship between subjective SES and risk aversion.

	Model 1			Model 2			Model 3		
Covariate	Estimate	SE	value of *p*	Estimate	SE	value of *p*	Estimate	SE	value of *p*
Age	0.034	0.016	0.032	0.034	0.016	0.033	0.034	0.016	0.033
Sex	−1.311	0.300	<0.0001	−1.302	0.306	<0.0001	−1.331	0.302	<0.0001
Race	0.811	0.283	0.004	0.815	0.282	0.004	0.682	0.308	0.027
Global cognition	−0.497	0.227	0.028	−0.498	0.228	0.029	−0.512	0.229	0.026
Education	−0.033	0.040	0.410	−0.033	0.040	0.410	−0.035	0.040	0.390
Income	−0.053	0.048	0.272	−0.053	0.048	0.271	−0.055	0.048	0.260
**Subjective SES**	**−0.238**	**0.083**	**0.004**	**−0.255**	**0.090**	**0.005**	**−0.190**	**0.095**	**0.046**
Subjective SES*Sex	–	–	–	0.095	0.204	0.641	–	–	–
Subjective SES*Race	–	–	–	–	–	–	−0.200	0.191	0.296

Males and Black participants are coded as 1; Females and non-Hispanic/Latino White participants are coded as 0; SE = standard error; SES = subjective socioeconomic status.

## Discussion

In this study, we investigated the relationship between subjective SES and risk aversion in a cohort of older adults without dementia. Consistent with our hypothesis, subjective SES was inversely associated with risk aversion independently of objective markers of SES such that those who rated themselves as having lower social standing relative to others in their community and in the United States demonstrated higher risk aversion. The relationship between subjective SES and risk aversion was not modified by sex or race. This finding builds on studies reporting an inverse association between objective indicators of SES and risk aversion across the adult lifespan (Riley and Chow, 1992; Grable, 2000; Bajtelsmit and Bernasek, 2001; Boyle et al., 2011; Kish-Gephart, 2017) and suggests that subjective impressions of SES may independently contribute to risk aversion. Findings support the importance of considering subjective indicators of SES in addition to objective indicators, as they may directly impact an older adult’s economic preferences.

To our knowledge, few studies have investigated contextual correlates of risk aversion in older adults. In previous work by our group (Boyle et al., 2011), we showed that lower global cognition was associated with more risk aversion, after adjustments for covariates including age, sex, education, and income. The present findings extend upon this study by showing that perceived social standing was associated with risk aversion in older adults without dementia after controlling for these same covariates, as well as race, and global cognition. Findings of this study add to the current knowledge base of contextual factors that are associated with risk aversion in older adults, by suggesting that a person’s perception of social standing may influence his or her willingness to take risk.

The association of subjective SES with risk aversion was significant after controlling for relevant covariates and objective indicators of SES (i.e., education, income). Additionally, education and income were not independently associated with risk aversion in the fully adjusted model. There are several possible reasons why subjective SES was found to be associated with risk aversion while objective markers of SES were not. Singh-Manoux et al. (2005) suggest that subjective SES may be a more precise measure of social status because it reflects an individual’s summary of different markers of SES and of past, present, and future expectations of social standing. Individuals may develop a more risk averse approach to financial decision making if they come from a past of lower SES or anticipate a future of economic hardships. Additionally, subjective SES may capture unique circumstances that objective indicators do not capture, such as variability in quality of education despite equivalent years of education (Operario et al., 2004; Singh-Manoux et al., 2005). Subjective SES also involves psychological components that objective measures cannot account for such as feelings of stress or anxiety (Operario et al., 2004) and a low sense of control (Sheehy-Skeffington, 2020) associated with low perceived social standing. Research has demonstrated that emotions such as anxiety and fear are associated with heightened risk aversion (Raghunathan and Pham, 1999; Lerner and Keltner, 2001; Han et al., 2007; Maner et al., 2007; Hartley and Phelps, 2012; Haushofer and Fehr, 2014). As such, subjective markers of SES may relate more robustly to risk aversion than objective markers of SES because they more directly capture negative affect associated with social standing.

Given the cross-sectional nature of these data, we are unable to determine causality. However, it is likely that risk aversion and low subjective SES are bidirectionally related and reinforcing of each other, a notion suggested in a review of poverty and economic risk preferences (Haushofer and Fehr, 2014). While low SES may lead to increased risk aversion, being more risk averse may result in lower SES by leading to economic behaviors that are disadvantageous (see Haushofer and Fehr, 2014). For example, Finke and Huston (2003) suggest that because less risk averse investors invest in more volatile financial instruments that are expected to have higher than average returns (e.g., stocks), they will accumulate higher than average net worth over time. Consistent with this, research has shown that risk aversion is associated with lower net worth and less retirement savings (Bernheim et al., 2001; Finke and Huston, 2003; Watson and McNaughton, 2007).

In this study, objective indicators of SES (education and income) were inversely correlated with risk aversion in bivariate analyses, though these relationships did not hold in the fully adjusted model. Few studies have investigated such associations specifically in older adult samples, as most studies have primarily focused on the effects of aging on risk aversion more generally (for discussion see our previous work, Boyle et al., 2011). Some studies that have examined associations between education and income and risk aversion in older adults have reported higher levels of education and income to be associated with less risk aversion (Barsky et al., 1997, Schurer, 2015 in regard to income), while others have not found evidence for such associations (Barsky et al., 1997 in regard to education), including a previous study by our group (Boyle et al., 2011). One possibility for the lack of association between objective indicators of SES and risk aversion in this study is that participants were highly educated (*M* years of education = 15.51, *SD* = 3.02) and reported high income levels (*M* income = 7.41, *SD* = 2.37), which may have impacted our ability to find associations between these variables and risk aversion. Another possibility is that relationships between education and income and risk aversion are dependent on the type of risk being examined. For example, Halek and Eisenhauer (2001) found the nature of relationships between demographic variables and risk aversion differ for pure risk (only losses are a possible outcome) vs. speculative risk (both losses and gains are possible outcomes). Future research is necessary to further understand the relationships between income and education and risk aversion in older adult samples.

We did not find that sex or race modified the relationship between subjective SES and risk aversion. This is in contrast to studies that have reported a differential relationship between subjective SES and health by sex (Demakakos et al., 2008) and race (Cundiff and Matthews, 2017). Given that this is the first study to our knowledge to investigate the relationship between subjective SES and risk aversion, more research is needed to further understand the role of sex and race on this relationship.

The findings of this study have some noteworthy implications. They support the importance of considering subjective indicators of SES when investigating correlates of financial risk aversion and other financial outcomes in older adults. Subjective measures of SES likely reflect important psychological processes or nuanced experiences that are not captured by objective measures. These psychological processes and experiences may directly impact an older adult’s economic preferences, which can in turn have an impact on health and wellbeing. Further research is needed to determine whether older adults with low subjective SES may benefit from instructional programs and informational tools that aid in understanding risk/benefit ratios of various financial and health decisions (our group made a similar suggestion for individuals with lower cognitive abilities; see Boyle et al., 2011).

There are some limitations that should be mentioned. First, this is a highly educated and predominantly female community-based sample of older adults. Although importantly there was a high percentage of Black participants in the sample. Examining associations between subjective SES and risk aversion in diverse groups is important for establishing the generalizability of these findings. Second, this is a cross-sectional study which limits our ability to comment on the directionality of these relationships. Third, other metrics of risk aversion such as occupational status or work experience that were unavailable in this study could further shed light on the relationship between subjective SES and risk aversion. On a similar note, one’s past experiences with risk taking may also impact his or her level of risk aversion and this may be a variable to consider in future studies of risk aversion in older adults. Finally, future studies may also consider examining these relationships in a cognitively healthy sample of older adults or stratifying samples by level of cognitive impairment, as certain factors (e.g., anxiety, depression) may impact judgments of individuals with mild cognitive impairment. This study also has notable strengths including utilization of a detailed assessment of risk aversion in a large and well-characterized sample of older adults without dementia.

## Concluding remarks

In a community-based cohort of 933 older adults, lower subjective SES was associated with greater financial risk aversion. Risk aversion has been associated with important financial outcomes (Bernheim et al., 2001; Finke and Huston, 2003; Watson and McNaughton, 2007) and decision making in older adults (Boyle et al., 2012). However, to our knowledge, this is one of only a handful of studies that examined contextual correlates of risk aversion in older adults. This study suggests that subjective SES is an important determinant of risk aversion, adding to the paucity of literature regarding factors associated with risk preferences in old age.

## Data availability statement

The datasets presented in this study can be found in online repositories. The names of the repository/repositories and accession number(s) can be found at: https://www.radc.rush.edu/.

## Ethics statement

The studies involving human participants were reviewed and approved by Rush University Institutional Review Board. The patients/participants provided their written informed consent to participate in this study.

## Author contributions

GW planned the study and the data analysis and wrote the paper. SH helped to plan the study and revise the paper. LY helped plan and supervise data analysis and revised the paper. LB, ML, and DB helped revise the paper. PB helped to plan the study and data analysis and revised the paper. All authors contributed to the article and approved the submitted version.

## Funding

This work was supported by the National Institute on Aging at the National Institutes of Health grants R01AG017917 to DB, R01AG033678 and R01AG060376 to PB, R01AG055430 to SH, and R01AG022018 to LB.

## Conflict of interest

The authors declare that the research was conducted in the absence of any commercial or financial relationships that could be construed as a potential conflict of interest.

## Publisher’s note

All claims expressed in this article are solely those of the authors and do not necessarily represent those of their affiliated organizations, or those of the publisher, the editors and the reviewers. Any product that may be evaluated in this article, or claim that may be made by its manufacturer, is not guaranteed or endorsed by the publisher.

## Footnotes

^1^ www.radc.rush.edu

^2^ https://macses.ucsf.edu/research/psychosocial/subjective.php
